# Ileal Endometriosis: A Rare Case

**DOI:** 10.7759/cureus.78413

**Published:** 2025-02-03

**Authors:** Soufiane Taibi, Abdelali Guellil, Kradi Yassin, Rachid Jabi, Mohammed Bouziane

**Affiliations:** 1 Department of General Surgery, Faculty of Medicine and Pharmacy, Laboratory of Anatomy, Microsurgery and Surgery Experimental and Medical Simulation, Mohamed VI University Hospital, Oujda, MAR

**Keywords:** crohn’s disease (cd), diagnostic laparoscopy, ileal endometriosis, intestinal stricture, surgical case reports

## Abstract

Endometriosis occurs when tissue similar to the lining of the uterus grows outside of it. Although it typically affects organs within the pelvis, it can sometimes spread to other areas in the abdomen. Involvement of the ileum is uncommon, and its symptoms can resemble those of conditions such as inflammatory and neoplastic disease. A 36-year-old woman with no significant medical history presented with abdominal pain, diarrhea, constipation, and vomiting. Imaging suggested a stenotic lesion in the terminal ileum, and colonoscopy was normal. After exploratory laparoscopy showed severe ileal stricture with fibrosis, a right colectomy was performed given the different diagnoses of these lesions, especially Crohn’s disease and neoplastic disease. Histopathology confirmed ileal endometriosis. This case emphasizes the diagnostic challenges of ileal endometriosis and the critical role of surgical intervention for accurate diagnosis and effective treatment.

## Introduction

Endometriosis is a common medical condition and a significant public health concern [1], in which ectopic endometrial tissue is found in extrauterine sites. While it commonly affects pelvic structures, it can spread to any abdominal organ and sometimes beyond [2]. The reported prevalence of bowel involvement in endometriosis varies widely, ranging from 3.8% to 37% of cases. Approximately 75% of intestinal endometriosis lesions are found in the rectum and sigmoid colon, while ileal involvement is exceptionally rare [3]. The diverse symptoms associated with ileal endometriosis often complicate its diagnosis, which can mimic other conditions such as Crohn’s disease or neoplasia. The diagnosis requires a high index of suspicion, particularly in atypical presentations, and often relies on a combination of imaging, endoscopic evaluation, and surgical exploration. However, in many cases, the definitive diagnosis is only achieved postoperatively through histopathological examination [4].

In this case report, we present a rare instance of ileal endometriosis in a 36-year-old woman with no endometriosis history who presented with symptoms unrelated to her menstrual cycle. Paraclinical examinations were unable to differentiate this case from Crohn’s disease, which was the most likely diagnosis. This report highlights the diagnostic complexities and underscores the importance of a multidisciplinary approach in managing such rare and challenging cases.

## Case presentation

**Figure 1:**
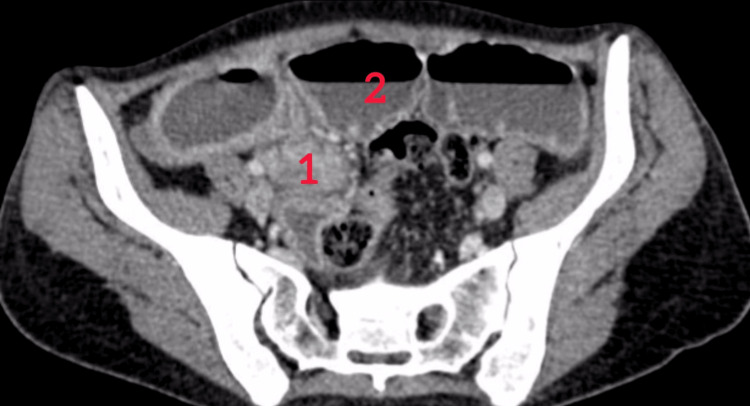
Axial CT image showing small bowel hydro-aeric distension below a stricture of the terminal ileum. (1) Ileal stricture. (2) Distension of the ileum.

**Figure 2:**
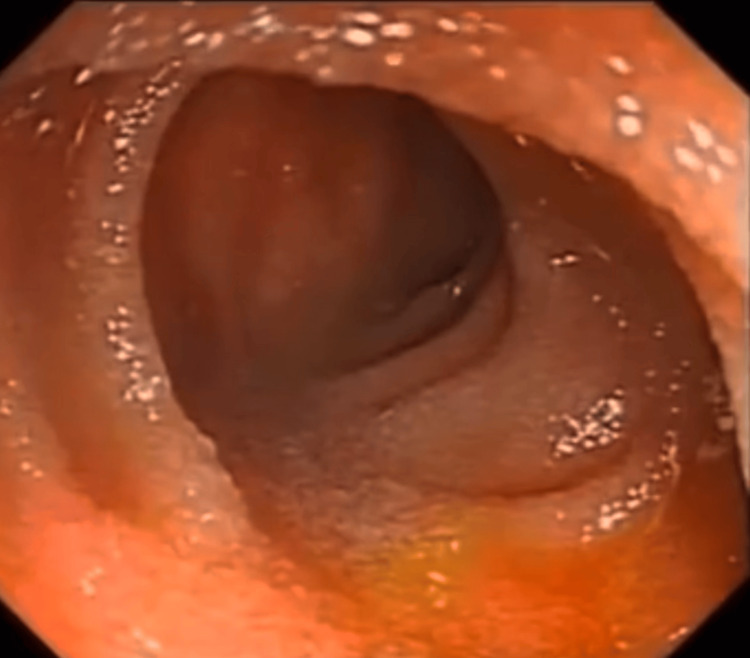
Colonoscopic image showing a normal ileal mucosa.

**Figure 3:**
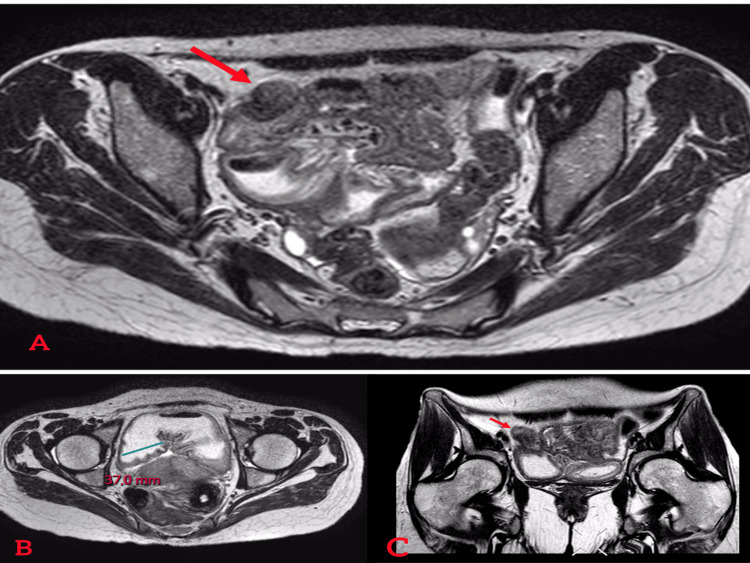
MRI findings. (A) Axial image showing an irregular and assymetric ileal stricture (arrow). (B) Terminal ileum distension. (C) Coronal image showing the ileal stricture (arrow).

A 36-year-old woman with no significant medical history such as endometriosis or any inflammatory disease presented with a five-day history of right iliac fossa abdominal pain. The patient’s last menstrual period was seven days before her admission. The pain was associated with alternating episodes of diarrhea and constipation, intermittent vomiting, and a preserved intestinal transit. She had no digestive bleeding or fever. These symptoms occurred within the context of general health decline. On clinical examination, she was conscious and hemodynamically stable. Abdominal examination revealed tenderness in the right iliac fossa, while rectal and vaginal examinations were unremarkable. Laboratory findings showed no significant abnormalities, except for hypochromic microcytic anemia with a hemoglobin of 8 g/dL (normal range: 12-16g/dL) and an elevated fecal calprotectin level of 621 µg/g (normal range <10 µg/g). A CT scan demonstrated significant hydro-aeric distension of small bowel loops upstream of a short, circumferential, irregular stenotic thickening in the terminal ileum, accompanied by peri-lesional fat infiltration. No rectosigmoid thickening was identified (Figure 1). Given this finding, chronic inflammatory bowel disease, Crohn’s type, was suspected. A colonoscopy revealed no abnormalities in the rectum and the mucosa of the terminal ileum (Figure 2). Further evaluation with MRI enterography confirmed findings of circumferential, irregular, and asymmetric thickening of the terminal ileum over a 15 cm segment, and no other thickening or strictures were noticed (Figure 3).

**Figure 4:**
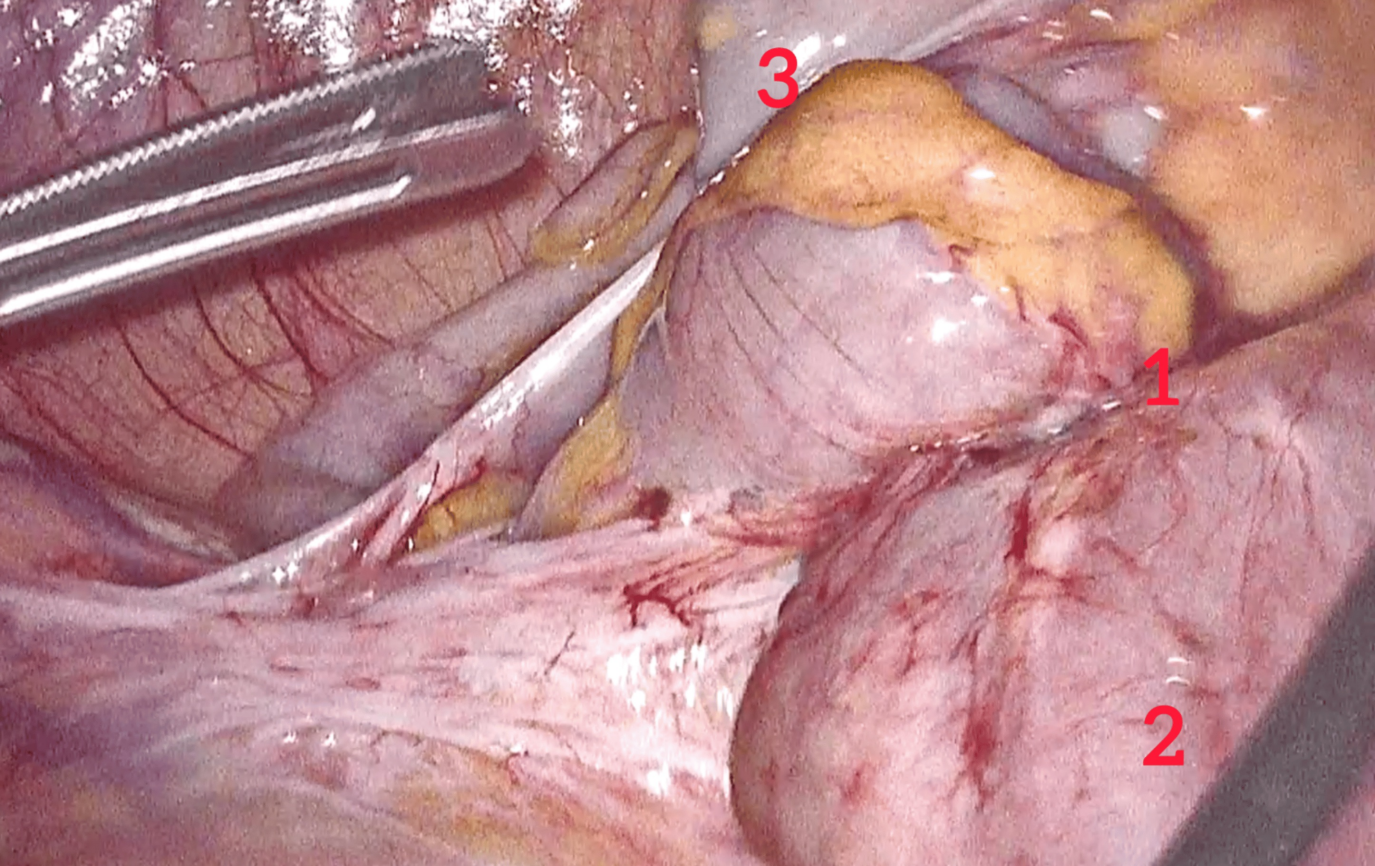
Laparoscopic image demonstrating fibrotic thickening of the ileal wall while the rest of abdominal cavity is normal. (1) Ileal stricture. (2) Terminal ileum. (3) Caecum.

**Figure 5:**
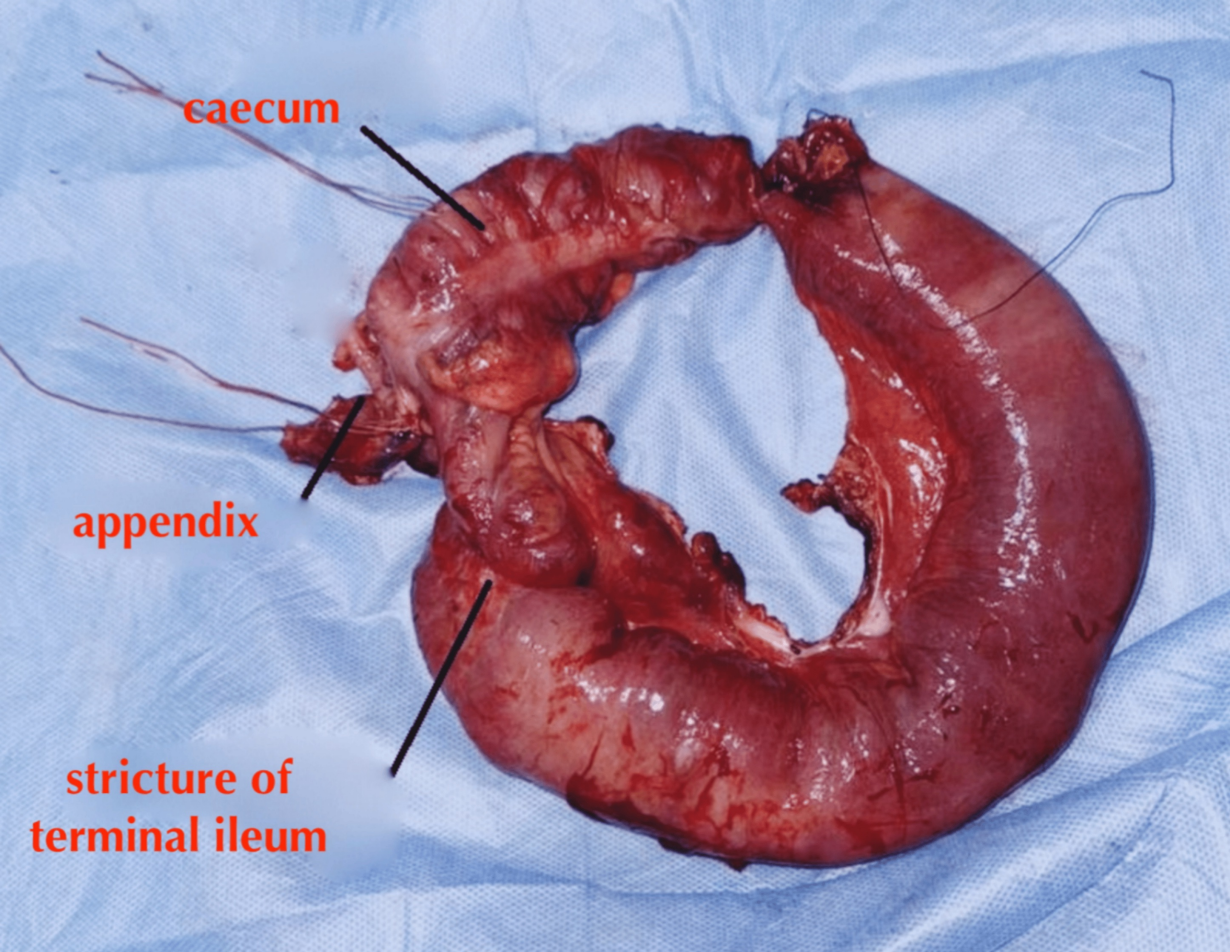
The resected spicemen (right colectomy).

**Figure 6:**
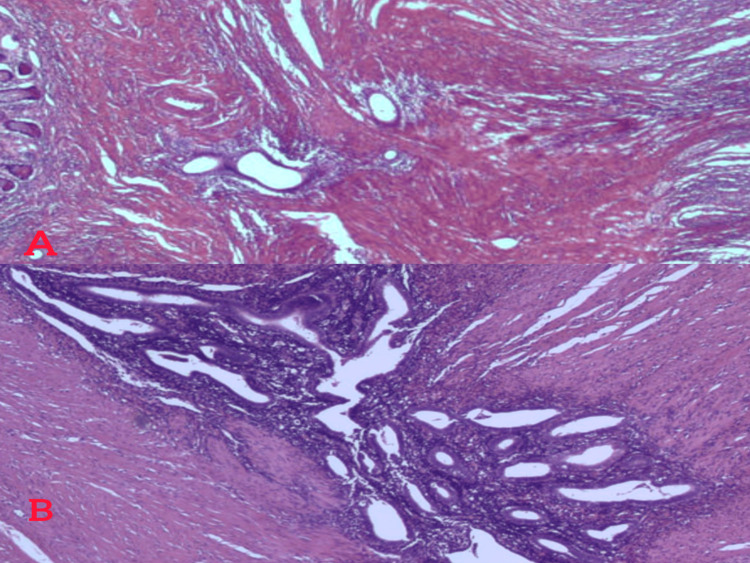
Histopathology findings. (A) Hematoxylin and eosin (×5) staining showing endometriosis of the ileum. (B) Hematoxylin and eosin staining (×10) showing glands and stroma of the endometriosis located in the wall of the terminal ileum.

After a multidisciplinary discussion, the decision was made to proceed with exploratory laparoscopy due to the suspicion of Crohn’s disease. Laparoscopy revealed fibrotic thickening and distension of the terminal ileum with no evidence of carcinomatosis or adenopathy. The remainder of the abdominal cavity appeared normal. A right colectomy with a side-to-side terminal anastomosis was performed (Figures 4, 5). Histopathological examination of the resected specimen confirmed the diagnosis of ileal endometriosis (Figure 6).

The postoperative course was uneventful, and the patient was referred to the gynecology department for further management and follow-up care.

## Discussion

Endometriosis is a chronic estrogen-dependent inflammatory condition defined by the presence outside the uterine cavity of endometrial-like tissue. It can spread to various abdominal organs and even beyond. Approximately 10% of reproductive-age women worldwide are affected by this condition, though the exact prevalence remains uncertain [5]. The involvement of the gastrointestinal tract in endometriosis was first described by Sampson in 1924. Estimates of bowel involvement vary widely, ranging between 3.8% and 37% of endometriosis cases. The rectosigmoid region is most commonly affected, accounting for 50-90% of intestinal lesions. In comparison, involvement of the small intestine is much rarer, with reported rates of 2-16% in the appendix, 3-18% in the cecum, and approximately 4.1% in the ileum [6,7]. Research on this topic has grown, especially in Europe and Asia, with the average age of patients reported at 40.3 years. [8]. The theory of retrograde menstruation is the most widely accepted explanation for the involvement of the digestive system in endometriosis. Other proposed mechanisms include the metaplastic transformation of pluripotent peritoneal mesothelium, the presence of Müllerian remnants, the spread of endometriosis cells along neural sheaths, and the role of genetic alterations [9]. When endometriotic tissue migrates to and settles on the bowel serosa, it may trigger a neovascularization process driven by vascular endothelial growth factors. This leads to continued growth and, in about 10% of cases, mucosal invasion. Repeated episodes can contribute to the development of fibrosis and smooth muscle hyperplasia [10]. Symptoms of bowel endometriosis are typically variable, depending on the location and depth of the implant’s penetration into the bowel wall. It is often diagnosed incidentally during surgery for asymptomatic cases, particularly in women without vaginal endometriosis, as seen in our case that was considered Crohn’s disease. While symptoms may be linked to the menstrual cycle in only 18-40% of cases, they can become persistent as the lesions progress under cyclical hormonal influences. This progression can cause the implants to proliferate in the bowel wall, leading to inflammation, fibrosis, and metaplasia or hyperplasia of the intestinal smooth muscles. Involvement of the serosa, submucosa, and mucosa may follow, resulting in symptoms such as abdominal pain, cramping, constipation, vomiting, and bleeding, particularly in cases affecting the rectum and sigmoid colon. This bowel infiltration can lead to complications such as luminal strictures and intestinal obstruction, making preoperative diagnosis of ileal endometriosis challenging. It may also mimic other conditions, such as Crohn’s disease (as in our case), ischemic colitis, or neoplasms [7-9]. If not properly treated, intestinal endometriosis can lead to life-threatening complications such as obstruction, perforation, hemorrhagic ascites, protein-losing enteropathy, anasarca, and intussusception. Although obstruction involving the small and large bowel is particularly rare, with a prevalence of 0.1-0.7%, other complications also remain uncommon [8-12].

Transvaginal ultrasonography should be the first-line investigation in women suspected of having rectosigmoid endometriosis. However, its benefit has not been proven in ileal endometriosis [13]. CT and MRI have similar sensitivities and demonstrate only moderate interobserver agreement. However, both are highly specific for diagnosing right-sided bowel deep infiltrating endometriosis. When used together, they complement each other, enhancing the detection of deep infiltrating endometriosis implants [14]. A colonoscopy should be performed systematically to rule out inflammatory diseases or malignant tumors arising from the intestinal mucosa [15].

Bowel endometriosis is mainly treated surgically, though balloon dilatation and stenting can be used in select cases. Pharmacological treatment has shown limited success, particularly in non-occlusive cases. Younger patients often have more aggressive disease. While endoscopic techniques are not standardized, most cases ultimately require surgery [8]. Surgery is indicated for patients with occlusive or subocclusive symptoms, those who do not respond to hormonal treatments, those with contraindications to hormonal therapy, or those with suspected malignancy or inflammatory disease to establish a diagnosis, as in our case. In cases of acute obstruction, radical resection may be required. Segmental resections, including right colectomy with side-to-side isoperistaltic with ileo-transverso-anastomosis, ileocecal resections, ileal resections, and ileo-transverse stomy, remain the gold standard for treating bowel endometriosis. These procedures are typically performed using laparoscopy or robotic-assisted laparoscopic surgery [16,17]. Given these considerations, and because we thought of Crohn’s disease, we chose to perform a diagnostic laparoscopy, which identified ileal involvement and led to a limited right hemicolectomy. The diagnosis was subsequently confirmed by histopathological examination.

## Conclusions

Ileal endometriosis, though rare, should be considered in premenopausal women presenting with intestinal obstruction symptoms, especially when mimicking conditions such as Crohn’s disease. This case highlights the diagnostic challenges, the critical role of surgical exploration, and the importance of histopathological confirmation. A multidisciplinary approach is essential for accurate diagnosis and effective management. Surgical resection is both diagnostic and therapeutic, offering relief for patients with severe symptoms or complications. This report underscores the need for greater awareness and improved diagnostic strategies to optimize outcomes for such atypical presentations.
